# Nutrient patterns and risk of diabetes mellitus type 2: a case-control study

**DOI:** 10.1186/s12902-024-01540-5

**Published:** 2024-01-17

**Authors:** Morteza haramshahi, Thoraya Mohamed Elhassan A-Elgadir, Hamid Mahmood Abdullah Daabo, Yahya Altinkaynak, Ahmed Hjazi, Archana Saxena, Mazin A.A. Najm, Abbas F. Almulla, Ali Alsaalamy, Mohammad Amin Kashani

**Affiliations:** 1 Faculty of medicine, Tabriz University of medical sciences, Tabriz, Iran; 2 Department of clinical biochemistry, College of medicine, King Khalid University, Abha, Saudi Arabia; 3 Fharmacy Department, Duhok polytechnic, University Duhok, Kurdistan, Iraq; 4 Department of Medical Services and Techniques, Ardahan University, Ardahan, Turkey; 5 Department of Medical Laboratory Sciences, College of Applied Medical Sciences, Prince Sattam bin Abdulaziz University, Jeddah, Saudi Arabia; 6 Department of Management, Uttaranchal Institute of Management, Uttaranchal University, Dehradun, Uttarakhand India; 7 Pharmaceutical Chemistry Department, College of Pharmacy, Al-Ayen University, Thi-Qar, Iraq; 8 College of technical engineering, The Islamic University, Najaf, Iraq; 9 College of technical engineering, Imam Ja’afar Al-Sadiq University, Al‐Muthanna, 66002 Iraq; 10 Department of Medicinal Chemistry, Faculty of Pharmacy, Tehran Medical Sciences, Islamic Azad University, Tehran, Iran

**Keywords:** Diabetes, T2D, Diet, Nutrient pattern

## Abstract

**Backgrounds:**

Although the significance of diet in preventing or managing diabetes complications is highlighted in current literature, there is insufficient evidence regarding the correlation between nutrient patterns and these complications. The objective of this case-control study is to investigate this relationship by analyzing the dietary intake of nutrients in participants with and without type 2 diabetes (T2D).

**Methods:**

A case-control study was conducted at the Tabriz Center of Metabolism and Endocrinology to investigate the relationship between nutrient patterns and type 2 diabetes (T2D). The study enrolled 225 newly diagnosed cases of T2D and 225 controls. The dietary intake of nutrients was assessed using a validated semi-quantitative food frequency questionnaire (FFQ). Principal component analysis using Varimax rotation was used to obtain nutrient patterns. Logistic regression analysis was performed to estimate the risk of T2D.

**Results:**

The participants’ mean (SD) age and BMI were 39.8 (8.8) years and 27.8 (3.6) kg/m2, respectively. The results identified three major nutrient patterns. The first nutrient pattern was characterized by high consumption of sucrose, animal protein, vitamin E, vitamin B1, vitamin B12, calcium, phosphorus, zinc, and potassium. The second nutrient pattern included fiber, plant protein, vitamin D, Riboflavin, Vitamin B5, copper, and Magnesium. The third nutrient pattern was characterized by fiber, plant protein, vitamin A, riboflavin, vitamin C, calcium, and potassium. Individuals in the highest tertile of nutrient pattern 3 (NP3) had a lower risk of T2D compared to those in the lowest tertile after adjusting for confounders. The odds ratio was 0.52 with a 95% confidence interval of 0.30–0.89 and a P_trend of 0.039.

**Conclusion:**

This study found that conforming to a nutrient pattern consisting of plant protein, vitamin C, vitamin A, vitamin B2, potassium, and calcium is linked to a lower likelihood of developing T2D.The initial results suggest that following a nutrient pattern that includes these nutrients may reduce the risk of T2D. However, further research is required to confirm the relationship between nutrient patterns and T2D.

## Background

Type 2 diabetes is a significant concern for public health in developed nations. It leads to high rates of illness and death and places a significant financial burden on healthcare systems [1, 2]. In the past few decades, there has been a sharp increase in the occurrence of diabetes, and is expected to continue increasing, with an estimated 693 million people living with the disease by 2045 [1]. Complications associated with type 2 diabetes can also contribute to premature death. A concerning aspect of the disease is that a significant proportion of cases (40%) go undetected [3], and there is also an increasing prevalence of prediabetes, which raises the risk of developing type 2 diabetes and other chronic diseases [1].

The connection between diet and type 2 diabetes has been extensively studied, including the examination of dietary patterns and individual foods or nutrient patterns [4–7]. Various sources have suggested that chronic diseases may be influenced by a combination of nutrients [8]. In the field of nutritional epidemiology, the examination of dietary patterns has emerged as a viable approach to investigate the correlation between diet and disease. This method involves using statistical techniques to combine multiple foods or nutrients into dietary or nutrient patterns, which are believed to provide a more detailed understanding of the connection between diet and disease. It has been suggested that the impact of individual nutrients or foods on chronic disease may be too subtle to detect, but their collective effect within a pattern may be more indicative [9].

There have been some recent studies examining the effect of nutrient patterns on chronic disease such as, non-alcoholic fatty liver, breast and gastric cancer, Polycystic Ovary Syndrome (PCOs) and metabolic syndrome [10–14]. For example, it was found that a nutrient pattern consisting mainly of protein, carbohydrates, and various sugars was linked to a higher risk of Metabolic Syndrome (MetS) in both men and women, whereas a pattern characterized by copper, selenium, and several vitamins was linked to greater odds of MetS [14]. A prospective study conducted among participants of the Tehran Lipid and Glucose Study indicates that a nutrient pattern rich in vitamin A, vitamin C, vitamin B6, potassium, and fructose is associated with a reduced risk of insulin-related disorders [15]. Although there have been limited investigations on the connection between nutrient patterns and the likelihood of developing diabetes, the present study seeks to explore this relationship by analyzing the adherence to different nutrient patterns and its effect on the risk of type 2 diabetes.

## Methods

### Study population

This study utilized a case-control design and involved participants between the ages of 18 and 60 who had been diagnosed with type 2 diabetes within the previous six months based on specific glucose level criteria (FBS levels of ≥ 126 mg/dl and 2 h-PG levels of ≥ 200 mg/dl [17]). Healthy individuals within the same age range were also included, with specific glucose level criteria (FBS levels of < 100 mg/dl and 2 h-PG levels of < 200 mg/dl [17]). The study excluded individuals with certain chronic diseases, Type 1 Diabetes, gestational diabetes, those following specific dietary patterns or taking certain medications, pregnant and breastfeeding women, those with a family history of diabetes or hypertension, and those who did not complete the food frequency questionnaire (more than 35 items) or whose reported energy intake was outside of a specific range (range of 800–4200 kcal [18]).

This study enrolled 450 adult participants, with 225 individuals in the case group and 225 in the control group. The case group was selected using a simple sampling method from patients diagnosed with diabetes at the Tabriz Center of Metabolism and Endocrinology as a referral center affiliated to tabriz University of Medical Sciences from January 2021 to March 2022, as well as through a two-stage cluster sampling method among patients referred to private endocrinologists to enhance the sample’s external validity. Participants in the control group were also selected through a two-stage cluster sampling method from individuals who had undergone blood glucose checkups at the Tabriz Center of Metabolism and Endocrinology, a referral center affiliated with Tabriz University of Medical Sciences, within the past six months. All participants provided informed consent at the beginning of the study. The study was financially supported by Tabriz University of Medical Sciences and is related to project NO. 1400/63,145.

### Dietary assessment

To collect dietary intake information, personal interviews and a semi-quantitative food frequency questionnaire (FFQ) consisting of 168 food items were used [16]. The FFQ asked about the frequency of consumption for each item over the course of one year, with the year before diagnosis for the case group and the year before the interview for the control group. Participants were also asked about the frequency of consumption (per day, week, month, or year) for each type of food. to ensure consistency in measurements, a nutritionist provided instructions on converting the size of reported food items from household measures to grams using four scales. The quantity of food consumed by each individual was calculated based on their intake in grams and reported on a daily basis. The nutrient composition of all foods was derived by using modified nutritionist IV software.

### Nutrient pattern assessment

We conducted factor analyses using a comprehensive set of 34 nutrients, encompassing various macronutrients, micronutrients, and other dietary components. These included sucrose, lactose, fructose, fiber, animal protein, plant protein, saturated fatty acids, monounsaturated fatty acids, polyunsaturated fatty acids, cholesterol, as well as an array of vitamins and minerals such as A, D, E, K, C, thiamine (B1), riboflavin (B2), niacin (B3), pantothenic acid (B5), pyridoxine (B6), folate (B9), B12, calcium, phosphorus, iron, zinc, copper, magnesium, manganese, chromium, selenium, sodium, potassium, and caffeine. The dietary intake of these 34 nutrients per 1,000 Kcal of energy intake was computed and utilized as input variables. Subsequently, nutrient patterns (NPs) were derived through principal component analysis (PCA) with varimax rotation, based on the correlation matrix. Factor scores for each participant were then calculated by aggregating the frequency of consumption and multiplying it by the factor loadings across all 34 nutrients. To assess the statistical correlation between variables and evaluate the adequacy of the sample size, we employed the Bartlett test of sphericity (*P* < 0.001) and the Kaiser-Mayer-Olkin test (0.71), respectively.

### Assessment of other variables

To obtain the participants’ anthropometric measurements, weight and height were measured using a seca scale, and the participants’ BMI was determined by dividing their weight in kilograms by the square of their height in meters. Waist circumference was measured using a metal anthropometric tape, and the participants’ hip circumference was measured using a metal anthropometric tape while standing [17]. Daily physical activity was measured using a physical activity questionnaire [18], and personal questioning was employed to gather information on population and socioeconomic characteristics, including marital status, academic degree, and smoking.

### Statistical analysis

Statistical analysis was performed using the Statistical Package Software for Social Science, version 21. The normality of the data was assessed using Kolmogorov-Smirnov’s test and histogram chart. The characteristics and dietary intakes of the case and control groups were presented as mean ± SD or median and frequency (percentages). Independent sample t-tests and chi-square tests were used to compare continuous and categorical variables, respectively, between the case and control groups.

## Results

The participants’ mean (SD) age and BMI were 39.8 (8.8) years and 27.8 (3.6) kg/m2, respectively. The mean (SD) BMI in the case group was 30.5 ± 4.1, and in the control group, it was 25.2 ± 3.2 kg/m2. The mean (SD) physical activity in the case group was 1121 ± 611 MET/min/week, and in the control group, it was 1598 ± 940 MET/min/week. There were significant differences in BMI and physical activity between the two groups. The mean (SD) waist circumference in the case group was 109.32 ± 10.28 cm, and in the control group, it was 87.25 ± 9.35 cm. The mean (SD) hip circumference in the case group was 107.25 ± 8.61 cm, and in the control group, it was 91.44 ± 6.17 cm. The study identified three primary nutrient patterns (NPs) with eigenvalues greater than 2. Table 1 displays the factor loadings for nutrient patterns, which accounted for 56.11% of the total nutrient variation. The high intake of sucrose, animal protein, phosphorus, zinc, potassium, calcium, vitamin E, vitamin B1 and vitamin B12 were the distinguishing features of the first pattern. The second nutrient pattern was positively associated with copper, magnesium, fiber, vitamin D, B2, B5 and plant protein but had a negative correlation with lactose and saturated fatty acids. On the other hand, the high intake of fiber, vitamin A, B2, vitamin C, plant protein and potassium were the distinguishing features of the third pattern.

**Table 1:** Factor loading matrix and explained variances for major nutrient patterns identified by factor analysis in 225 cases and 225 controls *^†^

Nutrient patterns				
Nutrients		Pattern 1	Pattern 2	Pattern 3
Animal protein		**0.67**		
Plant protein			**0.68**	**0.85**
Saturated fatty acids		0.36	**-0.48**	
Mono unsaturated fatty acids		-0.30		
Poly unsaturated fatty acids				
Cholesterol				
Sucrose		**0.91**		
Lactose			**-0.40**	
Fructose				
Fiber			**0.76**	**0.53**
Vitamin B1		**0.88**		-0.32
Vitamin B2			**0.71**	**0.46**
Vitamin B3				
Vitamin B5			**0.52**	
Vitamin B6				
Folate				
Vitamin B12		**0.49**		
Vitamin C				**0.79**
Vitamin A				**0.49**
Vitamin D			**0.78**	
Vitamin E		**0.63**		
Vitamin K				
Iron				
Zinc		**0.58**		0.35
Copper			**0.59**	0.31
Magnesium			**0.45**	
Manganese				
Calcium		**0.92**		**0.76**
Phosphor		**0.91**		
Sodium				
Potassium		**0.48**		**0.64**
**Explained variance (%)**		24.30	20.95	10.86
**Cumulative explained variance (%)**		24.30	45.25	56.11

^*^Principal Component Analysis performed on 34 nutrients calculated as intake per 1000 Kcal

Nutrients with loadings > 0.40 and less than − 0.40 (in bold) are being characteristic for the four patterns; loadings less than 0.3 (in absolute value) are suppressed

^†^Kaiser’s Measure of Sampling Adequacy, KMO = 0.71, Bartlett’s test of sphericity = < 0.001

The following are the characteristics of T2D patients compared to the control group, as shown in Table 2: Higher BMI, More likely to be smokers, Lower physical activity levels, higher FBS, HbA1C, Insulin (*p* < 0.05). Other variables did not differ significantly between the two groups (*p* > 0.05). Additionally, T2D patients had a greater intake of energy and vitamin B3 but consumed less plant protein, vitamin A, vitamin E, vitamin B2, and zinc (*p* < 0.05).

**Table 2:** Dietary intakes and Characteristics among cases and controls*

Demographic variables	Cases (*n* = 225)	Controls(*n* = 225)	***P***-value
Age(year)	39.6 ± 8.7	40.1 ± 8.9	0.206
Male, n (%)	119 (52.8)	112 (49.7)	0.347
BMI(Kg/m^2^)	30.5 ± 4.1	25.2 ± 3.2	< 0.001^a^
Smoking, n (%)	17 (7.5)	14 (6.2)	0.089
Physical activity (MET/min/week)	1121 ± 611	1598 ± 940	< 0.001^a^
SES, n (%)			0.274
Low	63 (28)	71 (31.5)	
Middle	106 (47.1)	101 (44.8)	
High	56 (24.9)	53 (23.5)	
FBS (mg/dl)	135.26 ± 14.52	91.26 ± 10.38	< 0.001^a^
HbA1C	8.6 ± 0.4	6.5 ± 0.1	< 0.001^a^
Insulin (mU/L)	26.23 ± 4.57	12.71 ± 0.98	0.02 ^a^
**Nutrient intake**			
Energy intake (Kcal/d)	2371 ± 624	2213 ± 619	0.008^a^
Sucrose(g/1000Kcal)	12.8 ± 6.4	14.2 ± 8.6	0.547
Lactose(g/1000Kcal)	6.5 ± 4.2	7.1 ± 4.5	0.332
Fructose(g/1000Kcal)	7.8 ± 3.3	7.9 ± 3.6	0.997
Fiber(g/1000Kcal)	16.2 ± 8.3	15.8 ± 6.5	0.218
Animal protein(g/1000Kcal)	22.4 ± 8.6	21.9 ± 8.5	0.072
Plant protein(g/1000Kcal)	13.8 ± 3.7	15.4 ± 3.9	0.024^a^
Saturated fatty acids(g/1000Kcal)	11.2 ± 3.4	11.5 ± 3.2	0.205
Mono unsaturated fatty acids(g/1000Kcal)	12.0 ± 3.3	11.9 ± 2.9	0.599
Poly unsaturated fatty acids(g/1000Kcal)	7.3 ± 2.8	7.1 ± 2.4	0.217
cholesterol(mg/1000Kcal)	97.4 ± 41.4	101.7 ± 56.0	0.057
Vitamin A(mg/1000Kcal)	186 ± 102	208 ± 110	0.009^a^
Vitamin D(µg/1000Kcal)	0.74 ± 0.55	0.95 ± 0.74	0.811
Vitamin E(mg/1000Kcal)	4.93 ± 1.50	5.12 ± 1.74	< 0.001^a^
Vitamin K(mg/1000Kcal)	78.5 ± 52.3	83.4 ± 57.2	0.214
Vitamin B1(mg/1000Kcal)	0.85 ± 0.18	0.81 ± 0.16	0.007^a^
Vitamin B2(mg/1000Kcal)	0.79 ± 0.20	0.86 ± 0.21	0.040 ^a^
Vitamin B3(mg /1000Kcal)	9.8 ± 2.3	9.6 ± 2.0	0.854
Vitamin B5(mg/1000Kcal)	2.32 ± 0.42	2.35 ± 0.44	0.147
Vitamin B6(mg/1000Kcal)	0.83 ± 0.16	0.81 ± 0.15	0.712
Folate(mg/1000Kcal)	236 ± 45	233 ± 38	0.625
Vitamin B12(mg/1000Kcal)	1.73 ± 0.86	1.79 ± 0.79	0.447
Vitamin C(mg/1000Kcal)	58.4 ± 31.1	60.2 ± 31.5	0.547
Calcium(mg/1000Kcal)	520 ± 163	525 ± 152	0.258
Phosphor(mg/1000Kcal)	621 ± 127	622 ± 119	0.741
Iron(mg/1000Kcal)	11.5 ± 5.4	11.4 ± 5.7	0.847
Zinc(mg/1000Kcal)	4.52 ± 0.80	5.44 ± 0.81	0.049 ^a^
Copper(mg/1000Kcal)	0.65 ± 0.13	0.69 ± 0.12	0.811
Magnesium(mg/1000Kcal)	157 ± 29	158 ± 30	0.784
Manganese(mg/1000Kcal)	3.2 ± 1.1	3.2 ± 1.0	0.974
Chromium(mg/1000Kcal)	0.04 (0.03–0.06)	0.04 (0.02–0.06)	0.414
Selenium(mg/1000Kcal)	48.5 ± 12.2	47.6 ± 11.7	0.532
Sodium(mg/1000Kcal)	1984 ± 1960	2022 ± 1385	0.736
Potassium(mg/1000Kcal)	1512 ± 312	1610 ± 381	0.033 ^a^
Caffeine(mg/1000Kcal)	54.4 ± 46.0	58.1 ± 44.8	0.355

*independent sample t-test and chi square

^a^*p* < 0.05

Table 3 summarizes the partial correlation coefficient between NPs and food sources, with NP1 showing a strong positive correlation with low-fat dairy, NP2 with refined grains, and NP3 with fruits and vegetables.

**Table 3:** Partial correlation coefficient of nutrient patterns with food sources^*^

	Nutrient pattern 1	Nutrient pattern 2	Nutrient pattern 3
Red and processed meat(g/d)	-0.025	-0.089^b^	-0.115^b^
White meats(g/d)	-0.048	-0.029	0.091
Plant oil (serving/d)	0.017	-0.018	0.115^b^
Nuts(g/d)	-0.024	-0.161^a^	0.247^a^
Low fat dairy(g/d)	0.712^a^	-0.165	0.034
High fat dairy(g/d)	0.244	-0.156^b^	-0.134^b^
Whole grain(g/d)	0.158^a^	-0.031	-0.087
Refined grain(g/d)	-0.242^a^	0.431^a^	-0.372^a^
Legume(g/d)	0.007	0.156^a^	-0.005
Fruits(g/d)	0.051	-0.082	0.847^a^
Vegetables(g/d)	0.089	0.038	0.673^a^
Egg (serving/d)	0.091^b^	0.003	0.038
Fruit juice (serving/d)	0.053	-0.079	0.366^a^
Snacks (serving/d)	-0.135	-0.281	-0.075^b^
Artificial beverages (serving/d)	-0.079^b^	-0.141^b^	0.074

^*^Adjusted for age, sex, and energy intake

^a^*P* < 0.001, ^b^*P* < 0.05

Table 4 demonstrates the relationships between NPs and T2D. After adjusting for age and sex, there was no significant link between each nutrient pattern (NP) and T2D. However, when adjusting for other factors such as BMI, physical activity, smoking, and energy intake, individuals in the highest tertile of NP1 and NP2 did not show a significant association with T2D compared to those in the lowest tertile. On the other hand, those in the highest tertile of NP3 had a lower probability of developing T2D than those in the lowest tertile (OR: 0.52, 95%CI: 0.30–0.89, P_trend = 0.039).

**Table 4:** Odds ratios (ORs) and 95% confidence intervals (CIs) for T2D based on tertiles of nutrient patterns

	Tertiles of nutrient patterns			***P*** for trend
	T1	T2	T3	
**Nutrient pattern 1**				
Median score	-0.94	-0.13	0.93	
T2D /control	78 / 72	78 / 72	69 / 81	
Model 1^*^	1.00 (Ref)	1.00 (0.71–1.50)	0.79 (0.51–1.20)	0.412
Model 2^†^	1.00 (Ref)	0.93 (0.55–1.57)	0.81 (0.49–1.41)	0.218
**Nutrient pattern 2**				
Median score	-0.91	-0.08	0.84	
T2D /control	73 / 76	73 / 75	79 / 74	
Model 1^*^	1.00 (Ref)	1.00 (0.59–1.38)	1.31 (0.89–1.92)	0.287
Model 2^†^	1.00 (Ref)	0.91 (0.48–1.41)	1.09 (0.54–1.67)	0.850
**Nutrient pattern 3**				
Median score	-0.96	-0.13	0.99	
T2D /control	75 / 75	84 / 77	66 / 73	
Model 1^*^	1.00 (Ref)	1.12 (0.81–1.61)	0.94 (0.61–1.34)	0.387
Model 2^†^	1.00 (Ref)	0.87 (0.50–1.49)	0.52 (0.30–0.89)	0.039

^*^Model 1: Adjusted for age and sex

^†^Model 2: Additionally adjusted for model 1 and BMI, physical activity, smoking, dietary intake of energy

## Discussion

In this study, three major NPs were identified. After adjusting for potential confounders, we observed a significant inverse association between the Third NP and the odds of T2D. The high intake of fiber, vitamin A, B2, vitamin C, plant protein and potassium were the distinguishing features of the third pattern.

Dietary patterns, such as healthy, Mediterranean, traditional, and Western dietary patterns, have recently received significant attention in studying the connection between diet and health. When looking at the relationship between nutrients and disease incidence, it is more challenging to evaluate when considering individual foods and the metabolism of all nutrients together [19]. It is therefore more effective to take a broader view and consider diet as a whole. Dietary and nutrient patterns can have a greater impact on health than specific nutrients or nutritional groups. There is supporting evidence that links high calorie or high glycemic index foods with an increased risk of T2D. The quality of one’s diet is also associated with the risk, progression, and side effects of T2D [20]. Establishing a desirable food pattern has become a priority in public health efforts to prevent T2D. By studying dietary and nutrient patterns, we can gain a comprehensive understanding of an individual’s overall diet beyond just the consumption of specific nutrients and food groups. Moreover, it is easier for people to understand health recommendations when presented as dietary patterns rather than focusing solely on individual nutrients [19].

A previous cross-sectional study investigated the relationship between NPs and fasting glucose and glycated hemoglobin levels among apparently healthy black South Africans. The study stratified 2,010 participants by gender and urban/rural status and identified three nutrient patterns per stratum. In rural women, a nutrient pattern driven by starch, dietary fiber, and B vitamins was significantly associated with lower fasting glucose and glycated hemoglobin levels. A nutrient pattern that included vitamin B1, zinc, and plant protein was linked to notable decreases in glycated hemoglobin and fasting glucose levels in rural men. These findings suggest that nutrient patterns that are plant-based are linked to lower levels of fasting glucose and glycated hemoglobin [21].

Iwasaki et al. found that specific nutrient patterns were associated with lower risks of MetS. One nutrient pattern high in potassium, fiber, and vitamins, while another pattern high in vitamin B2, saturated fatty acids and calcium [22]. A recent study found that a nutrient pattern characterized by high intake of calcium, potassium, fats, cholesterol, vitamins B2, B12, A, D, K and C was positively linked to MetS [23]. Salehi-Sahlabadi et al. found that adhering to a nutrient pattern rich in potassium, vitamin A, fructose, vitamin C and vitamin B6 was negatively associated with the likelihood of NAFLD [11]. A nutrient pattern high in potassium, vitamin A, vitamin B6, vitamin C and fructose was associated with a reduced risk of hyperinsulinemia, IR, and dyslipidemia among participants in Tehran, according to a prospective study [11, 24, 25].

Due to several variations among studies exploring NPs linked to chronic diseases, including differences in the number of nutrients, populations, study designs and outcomes there has been a considerable diversity in the identified NPs, with only a few NPs being replicated across studies. Our study is the first of its kind to explore the correlation between nutrient patterns and T2D in this context.

In our study, there was no association between NPs 1 and 2 and T2D. This lack of correlation may be attributed to the absence of harmful nutrients or food categories linked to diabetes in these NPs. NP3 in this study, unlike other NPs, is positively associated with beneficial food groups such as nuts, fruits, plant oil and vegetables, and negatively associated with unhealthy food groups like red-processed meat, snacks, high-fat dairy and refined grains. A recent systematic review and meta-analysis found that individuals who consumed higher amounts of fruits and vegetables had a lower risk of developing type 2 diabetes [26]. Moreover, the consumption of vegetables was found to have an inverse relationship with ALT, TC and LDL levels among adults, while fruit consumption was associated with a positive reduction in visceral fat [27, 28]. Another study suggested that an increased intake of vegetables and fruits could potentially lower the risk of MetS [29]. According to a study, greater nut consumption was significantly linked to a reduced prevalence of T2D [30]. Consuming fruits and vegetables is a crucial component of a healthful dietary pattern that can lower the risk of type 2 diabetes [31]. On the other hand, Consuming a Western dietary pattern, which primarily consists of fast foods, high-fat dairy, refined grains, soft drinks and processed meat has been found to be correlated with an increased risk of type 2 diabetes [31].

Several mechanisms have been identified that explain the positive associations between the components of NP 3 and T2D or its risk factors. Vitamin intake has been shown to play a role in the development of T2D through various pathways. Consuming vitamin C has been found to have beneficial effects in reducing the risk of type 2 diabetes mellitus. These effects can be attributed to the following actions of vitamin C: vasodilator, cytoprotective, platelet anti-aggregator and anti-mutagenic. To achieve this, the body increases the production of several substances including prostaglandin E1, PGI2, endothelial nitric oxide, and lipoxin A4. Additionally, the body restores the Arachidonic Acid content to normal levels [32]. Vitamin A has a multifaceted role in cell regulation beyond its antioxidant function. It contributes to gene regulation, epithelial cell integrity, and resistance to infection. Research suggests that vitamin A also enhances antioxidant enzyme function in the body. Research has indicated a link between vitamin A deficiency and type 2 diabetes mellitus (T2DM), which suggests that vitamin A may have a role in the biology of T2DM [33]. Moreover, a meta-analysis has found that replacing animal protein with plant protein can lead to minor improvements in glycemic control for individuals with diabetes [34]. According to a recent meta-analysis, increasing the consumption of fruits, especially berries, yellow vegetables, cruciferous vegetables, green leafy vegetables is associated with a lower risk of developing type 2 diabetes. These results support the recommendation to incorporate more fruits and vegetables into the diet as a way to prevent various chronic diseases, including type 2 diabetes [35]. A study showed that maintaining adequate potassium intake could regulate insulin secretion and carbohydrate metabolism, leading to the prevention of obesity and metabolic syndrome (MetS) [36].

A number of research studies conducted in the Western societies have shown that Western dietary pattern including higher intake of red meat, processed meat, and refined grains is significantly associated with increased risk of T2D [37, 38]. For example, in the 12-years cohort prospective study, van Dam et al. investigated dietary pattern of 42,504 American white men at the age range of 40–75 years old using the FFQ. After controlling the confounders, the risk of T2D increased 60% in people adherent to the western-like dietary pattern [38]. The rapid process of change in lifestyle, diets, and physical activity that have been occurred as a result of extended urbanization, improved economic status, change of work pattern toward jobs, and change in the processes of producing and distributing nutrients during the recent years in developing countries have led people to more consumption of fast food and processed foods [20].

Significant research has been conducted on the impact of nutrient type and sequence on glucose tolerance. Multiple studies have shown that manipulating the sequence of food intake can enhance glycemic control in individuals with type 2 diabetes in real-life situations. The glucose-lowering effect of preload-based nutritional strategies has been found to be more pronounced in type 2 diabetes patients compared to healthy individuals. Moreover, consuming carbohydrates last, as part of meal patterns, has been proven to improve glucose tolerance and reduce the risk of weight gain [39]. Recent findings on meal sequence further emphasize the potential of this dietary approach in preventing and managing type 2 diabetes [40].

Several studies have shown that food from a short supply chain has a significant impact on metabolic syndrome. The length of the food supply chain is important in determining the risk of metabolic syndrome in a population [41]. Research indicates that people who consume food from short supply chains have a lower prevalence of metabolic syndrome compared to those who consume food from long supply chains. Specifically, food from short supply chains is associated with lower levels of triglycerides and glucose, which leads to a reduced occurrence of metabolic syndrome [42]. Adhering to the Mediterranean diet with a short supply chain is also found to significantly reduce the prevalence of metabolic syndrome. Therefore, these studies provide evidence that food from short supply chains positively affects metabolic parameters and the occurrence of metabolic syndrome [41].

The study we conducted presented several advantages. It was the first case-control research to investigate the correlation between nutrient patterns and the likelihood of developing type 2 diabetes (T2D). While numerous studies have explored the relationship between dietary patterns and diabetes, there is a scarcity of research specifically focusing on nutrient patterns in individuals with type 2 diabetes. Furthermore, the collection of dietary intake data was carried out through face-to-face interviews conducted by trained dieticians to minimize measurement errors. However, this study also had some limitations. Case-control studies are susceptible to selection and recall biases. Additionally, the use of factor analysis to identify patterns, and the potential influence of research decisions on the number of factors and nutrient factor loadings in each pattern, should be considered. Lastly, despite the use of a validated semi-quantitative FFQ (food frequency questionnaire), there remains a possibility of measurement error due to dietary recall. The study’s findings and limitations contribute to the ongoing discourse on the role of nutrient patterns in the development of T2D and the importance of considering these factors in future research and preventive strategies.

## Conclusions

The results of this study indicate that conforming to a nutrient pattern consisting of plant protein, vitamin C, vitamin A, vitamin B2, potassium, and calcium is linked to a lower likelihood of developing T2D. Our investigation did not reveal any significant correlation between other nutrient patterns and T2D risk. However, additional research is necessary to authenticate these initial findings and establish the correlation between nutrient patterns and T2D.

## Data Availability

Upon reasonable request, the corresponding author can provide the datasets that were produced and analyzed during the current study.

## References

[1] Ogurtsova K, Guariguata L, Barengo NC, Ruiz PL-D, Sacre JW, Karuranga S (2022). IDF Diabetes Atlas: global estimates of undiagnosed diabetes in adults for 2021. Diabetes Res Clin Pract.

[2] Teo ZL, Tham Y-C, Yu M, Chee ML, Rim TH, Cheung N (2021). Global prevalence of diabetic retinopathy and projection of burden through 2045: systematic review and meta-analysis. Ophthalmology.

[3] Sugiyama T, Yanagisawa-Sugita A, Tanaka H, Ihana-Sugiyama N, Imai K, Ohsugi M (2023). Different incidences of diabetic retinopathy requiring treatment since diagnosis according to the course of diabetes diagnosis: a retrospective cohort study. Sci Rep.

[4] Hodge AM, English DR, O’Dea K, Giles GG (2007). Dietary patterns and diabetes incidence in the Melbourne Collaborative Cohort Study. Am J Epidemiol.

[5] Jannasch F, Kröger J, Schulze MB (2017). Dietary patterns and type 2 diabetes: a systematic literature review and meta-analysis of prospective studies. J Nutr.

[6] Sami W, Ansari T, Butt NS, Ab Hamid MR (2017). Effect of diet on type 2 diabetes mellitus: a review. Int J Health Sci.

[7] Miller V, Micha R, Choi E, Karageorgou D, Webb P, Mozaffarian D (2022). Evaluation of the quality of evidence of the association of foods and nutrients with cardiovascular disease and diabetes: a systematic review. JAMA Netw open.

[8] Salehi-Sahlabadi A, Teymoori F, Jabbari M, Momeni A, Mokari-Yamchi A, Sohouli M (2021). Dietary polyphenols and the odds of non-alcoholic fatty liver disease: a case-control study. Clin Nutr ESPEN.

[9] Salehi-Abargouei A, Esmaillzadeh A, Azadbakht L, Keshteli AH, Feizi A, Feinle-Bisset C (2016). Nutrient patterns and their relation to general and abdominal obesity in Iranian adults: findings from the SEPAHAN study. Eur J Nutr.

[10] Panjeshahin A, Salehi-Abargouei A, Ghadiri-Anari A, Rasouli A, Hosseinzadeh M. The Association between nutrient patterns and polycystic ovary syndrome: a case-control study. J Nutr Food Secur. 2022. 10.1016/j.nut.2020.110987 32947130

[11] Salehi-Sahlabadi A, Teymoori F, Ahmadirad H, Mokhtari E, Azadi M, Seraj SS (2022). Nutrient patterns and non-alcoholic fatty liver disease in Iranian Adul: a case-control study. Front Nutr.

[12] Fereidani SS, Eini-Zinab H, Heidari Z, Jalali S, Sedaghat F, Rashidkhani B (2018). Nutrient patterns and risk of breast cancer among Iranian women: a case-control study. Asian Pac J cancer Prevention: APJCP.

[13] Narmcheshm S, Sasanfar B, Hadji M, Zendehdel K, Toorang F, Azadbakht L (2022). Patterns of nutrient intake in relation to gastric cancer: a case control study. Nutr Cancer.

[14] Khayyatzadeh SS, Moohebati M, Mazidi M, Avan A, Tayefi M, Parizadeh SMR (2016). Nutrient patterns and their relationship to metabolic syndrome in Iranian adults. Eur J Clin Invest.

[15] Teymoori F, Mokhtari E, Salehi P, Hosseini-Esfahani F, Mirmiran P, Azizi F (2021). A nutrient pattern characterized by vitamin A, C, B6, potassium, and fructose is associated with reduced risk of insulin-related disorders: a prospective study among participants of Tehran lipid and glucose study. Diabetol Metab Syndr.

[16] Esmaillzadeh A, Azadbakht L (2008). Major dietary patterns in relation to general obesity and central adiposity among Iranian women. J Nutr.

[17] Wang J, Thornton JC, Bari S, Williamson B, Gallagher D, Heymsfield SB (2003). Comparisons of waist circumferences measured at 4 sites. Am J Clin Nutr.

[18] Committee IR. Guidelines for data processing and analysis of the International Physical Activity Questionnaire (IPAQ)-short and long forms. http://www.ipaq.ki.se/scoring.pdf; 2005.

[19] Mandalazi E, Drake I, Wirfält E, Orho-Melander M, Sonestedt E (2016). A high diet quality based on dietary recommendations is not associated with lower incidence of type 2 diabetes in the Malmö Diet and Cancer Cohort. Int J Mol Sci.

[20] Beigrezaei S, Ghiasvand R, Feizi A, Iraj B (2019). Relationship between dietary patterns and incidence of type 2 diabetes. Int J Prev Med.

[21] Chikowore T, Pisa PT, Van Zyl T, Feskens EJ, Wentzel-Viljoen E, Conradie KR (2017). Nutrient patterns associated with fasting glucose and glycated haemoglobin levels in a black South African population. Nutrients.

[22] Iwasaki Y, Arisawa K, Katsuura-Kamano S, Uemura H, Tsukamoto M, Kadomatsu Y (2019). Associations of nutrient patterns with the prevalence of metabolic syndrome: results from the baseline data of the Japan multi-institutional collaborative cohort study. Nutrients.

[23] Sadeghi O, Sadeghi A, Mozaffari-Khosravi H, Shokri A (2021). The association between nutrient patterns and metabolic syndrome among Iranian adults: cross-sectional analysis of Shahedieh cohort study. Public Health Nutr.

[24] Mottaghian M, Salehi P, Teymoori F, Mirmiran P, Hosseini-Esfahani F, Azizi F (2020). Nutrient patterns and cardiometabolic risk factors among Iranian adults: Tehran lipid and glucose study. BMC Public Health.

[25] Teymoori F, Mokhtari E, Salehi P, Hosseini-Esfahani F, Mirmiran P, Azizi F (2021). A nutrient pattern characterized by vitamin A, C, B6, potassium, and fructose is associated with reduced risk of insulin-related disorders: a prospective study among participants of Tehran lipid and glucose study. Diabetol Metab Syndr.

[26] Halvorsen RE, Elvestad M, Molin M, Aune D (2021). Fruit and vegetable consumption and the risk of type 2 diabetes: a systematic review and dose–response meta-analysis of prospective studies. BMJ Nutr Prev Health.

[27] Mollahosseini M, Daneshzad E, Rahimi MH, Yekaninejad MS, Maghbooli Z, Mirzaei K (2017). The association between fruit and vegetable intake and liver enzymes (aspartate and alanine transaminases) in Tehran, Iran. Ethiop J Health Sci.

[28] Plaz Torres MC, Bodini G, Furnari M, Marabotto E, Zentilin P, Giannini EG (2020). Nuts and non-alcoholic fatty liver disease: are nuts safe for patients with fatty liver disease?. Nutrients.

[29] Lee M, Lim M, Kim J (2019). Fruit and vegetable consumption and the metabolic syndrome: a systematic review and dose–response meta-analysis. Br J Nutr.

[30] Muley A, Fernandez R, Ellwood L, Muley P, Shah M (2021). Effect of tree nuts on glycemic outcomes in adults with type 2 diabetes mellitus: a systematic review. JBI Evid Synthesis.

[31] Beigrezaei S, Ghiasvand R, Feizi A, Iraj B. Relationship between dietary patterns and incidence of type 2 diabetes. Int J Prev Med. 2019;10. 10.4103/ijpvm.IJPVM_206_17 PMC6639850 31367285

[32] Das UN (2019). Vitamin C for type 2 diabetes mellitus and hypertension. Arch Med Res.

[33] Iqbal S, Naseem I (2015). Role of vitamin A in type 2 diabetes mellitus biology: effects of intervention therapy in a deficient state. Nutrition.

[34] Viguiliouk E, Stewart SE, Jayalath VH, Ng AP, Mirrahimi A, De Souza RJ (2015). Effect of replacing animal protein with plant protein on glycemic control in diabetes: a systematic review and meta-analysis of randomized controlled trials. Nutrients.

[35] Wang PY, Fang JC, Gao ZH, Zhang C, Xie SY (2016). Higher intake of fruits, vegetables or their fiber reduces the risk of type 2 diabetes: a meta-analysis. J Diabetes Invest.

[36] Cai X, Li X, Fan W, Yu W, Wang S, Li Z (2016). Potassium and obesity/metabolic syndrome: a systematic review and meta-analysis of the epidemiological evidence. Nutrients.

[37] van Dam RM, Rimm EB, Willett WC, Stampfer MJ, Hu FB (2002). Dietary patterns and risk for type 2 diabetes mellitus in US men. Ann Intern Med.

[38] Fung TT, Schulze M, Manson JE, Willett WC, Hu FB (2004). Dietary patterns, meat intake, and the risk of type 2 diabetes in women. Arch Intern Med.

[39] Wheeler ML, Dunbar SA, Jaacks LM, Karmally W, Mayer-Davis EJ, Wylie-Rosett J (2012). Macronutrients, Food groups, and eating patterns in the management of diabetes: a systematic review of the literature, 2010. Diabetes Care.

[40] Dwibedi C, Mellergård E, Gyllensten AC, Nilsson K, Axelsson AS, Bäckman M (2022). Effect of self-managed lifestyle treatment on glycemic control in patients with type 2 diabetes. Npj Digit Med.

[41] Santulli G, Pascale V, Finelli R, Visco V, Giannotti R, Massari A (2019). We are what we eat: impact of food from short supply chain on metabolic syndrome. J Clin Med.

[42] De Rosa M, Giannotti R, Pascale A, Finelli R, Ilario M, Ciccarelli M (2018). P6280 food of short supply chain impacts metabolism and cardiovascular risk. A survey in Southern Italy. Eur Heart J.

